# New Target for Precision Medicine Treatment of Giant-Cell Tumor of Bone: Sunitinib Is Effective in the Treatment of Neoplastic Stromal Cells with Activated PDGFRβ Signaling

**DOI:** 10.3390/cancers13143543

**Published:** 2021-07-15

**Authors:** Michal Mahdal, Jakub Neradil, Peter Mudry, Silvia Paukovcekova, Iva Staniczkova Zambo, Jiri Urban, Peter Macsek, Lukas Pazourek, Tomas Tomas, Renata Veselska

**Affiliations:** 1 First Department of Orthopedic Surgery, St. Anne’s University Hospital and Faculty of Medicine, Masaryk University, 65691 Brno, Czech Republic; michal.mahdal@fnusa.cz (M.M.); lukas.pazourek@fnusa.cz (L.P.); tomas.tomas@fnusa.cz (T.T.); 2 Laboratory of Tumor Biology, Department of Experimental Biology, Faculty of Science, Masaryk University, 61137 Brno, Czech Republic; jneradil@sci.muni.cz (J.N.); silvia.paukovcekova@mail.muni.cz (S.P.); macsek@mail.muni.cz (P.M.); 3 International Clinical Research Center, St. Anne’s University Hospital, 65691 Brno, Czech Republic; mudry.peter@fnbrno.cz (P.M.); iva.zambo@fnusa.cz (I.S.Z.); 4 Department of Pediatric Oncology, University Hospital Brno and Faculty of Medicine, Masaryk University, 66263 Brno, Czech Republic; 5 First Pathology Department, St. Anne’s University Hospital and Faculty of Medicine, Masaryk University, 65691 Brno, Czech Republic; 6 Department of Chemistry, Faculty of Science, Masaryk University, 61137 Brno, Czech Republic; urban@chemi.muni.cz

**Keywords:** giant-cell tumor of bone, targeted treatment, sunitinib, PDGFR beta, denosumab, signaling

## Abstract

**Simple Summary:**

The purpose of this study was to analyze differential cell signaling in response to denosumab treatment to identify and subsequently inhibit molecular targets in the neoplastic stromal cell population, which poses a risk for tumor recurrence. Using phosphoprotein arrays, a distinct signaling profile was detected in GCTB tissues treated with denosumab, a specific RANKL antibody, which coincided with the RTK profile in derived cell lines. PDGFRβ was selected as a promising receptor target, and its inhibition by the small-molecule inhibitor sunitinib resulted in potent inhibition of cell proliferation *in vitro*. The addition of sunitinib to denosumab resulted in the disappearance of both multinuclear giant cells and neoplastic stromal cells, as reported here. Thus, sunitinib could become an effective addition to denosumab in the treatment of GCTB with activated PDGFRβ.

**Abstract:**

Giant-cell tumor of bone (GCTB) is an intermediate type of primary bone tumor characterized by locally aggressive growth with metastatic potential. The aim of this study was to identify new druggable targets among the cell signaling molecules involved in GCTB tumorigenesis. Profiles of activated signaling proteins in fresh-frozen tumor samples and tumor-derived cell lines were determined using phosphoprotein arrays. Analysis of the obtained data revealed epidermal growth factor receptor (EGFR) and platelet-derived growth factor receptor beta (PDGFRβ) as potential targets, but only the PDGFR inhibitor sunitinib caused a considerable decrease in stromal cell viability *in vitro*. Furthermore, in the case of a 17-year-old patient suffering from GCTB, we showed that the addition of sunitinib to the standard treatment of GCTB with the monoclonal antibody denosumab resulted in the complete depletion of multinucleated giant cells and mononuclear stromal cells in the tumor tissue. To summarize, the obtained data showed that a specific receptor tyrosine kinase (RTK) signaling pattern is activated in GCTB cells and plays an important role in the regulation of cell proliferation. Thus, activated RTKs and their downstream signaling pathways represent useful targets for precision treatment with low-molecular-weight inhibitors or with other types of modern biological therapy.

## 1. Introduction

GCTB is an intermediate type of primary bone neoplasia characterized by locally aggressive growth and low metastatic potential [1,2]. GCTB represents approximately 5–10% of all primary bone tumors [3,4], and it usually develops in adults between the ages of 20 and 40 years. Most cases occur around the knee, followed by the distal end of the radius [1,3,5]. Depending on the type of treatment and the local presentation of the tumor, recurrence rates vary from 33% to 65% after curettage and resection [1,5,6]. The frequency of lung metastases ranges from 2% to 5%, and the risk for the development of lung metastases seems to be associated with local recurrence [7,8].

Clinical symptoms include pain, local swelling, and limited range of motion of the adjacent joint. Pathological fractures may occur in approximately 10–12% of patients at diagnosis. The presence of a pathological fracture is associated with a higher risk of local recurrence [3,9]. From a histological point of view, GCTB contains two groups of cells: osteoclast-like giant cells and mononuclear (stromal) cells. Stromal cells express receptor activator of nuclear factor kappa B ligand (RANKL), and via the RANKL-RANK pathway, they activate osteoclasts. Osteoclast-like giant cells are secondarily recruited into the tumor and are responsible for its aggressive osteolytic activity [10,11]. Campanacci classified GCTB into three grades according to their radiographic appearance: Grade 1 lesions have a well-defined margin and an intact cortex, Grade 2 lesions have a relatively well-defined margin but no radiopaque rim, and the cortical bone is thinned and Grade 3 lesions have indistinct borders with cortical destruction and soft tissue extension.

Surgery remains the primary treatment modality for giant-cell tumors of bone. Curettage alone has been the standard treatment for GCTB, but it has been associated with a high risk of local recurrence. To reduce this risk of local recurrence, various local adjuvant therapies, such as cryosurgery, phenol, bone cement and argon beam application, as well as systemic treatment using bisphosphonates, interferon alpha (IFN-α) or denosumab, have been reported [5,12,13]. *En bloc* resection should be considered in the case of recurrent or locally advanced disease [9,14,15]. Denosumab is a monoclonal human antibody against RANKL that inhibits osteoclast-like giant cells but does not affect mononuclear tumor cells [11,14,15]. The use of denosumab is indicated in cases when radical resection cannot be performed [12]. Denosumab has also been successfully used to control metastatic lung disease [16]. However, the question remains as to how long denosumab can be used and what side effects it may have. Denosumab withdrawal is associated with a high rate of subsequent tumor recurrence. Treatment with denosumab resulted in the disappearance of all osteoclast-like giant cells in GCTB tumor tissue but had no effect on neoplastic stromal cells, which persist and continue to proliferate [17].

The cellular and molecular mechanisms controlling disease progression in GCTB remain unclear. Suggested biomarkers associated with GCTB disease progression include increased expression of human telomerase reverse transcriptase, p53, c-Myc oncogene, matrix metalloproteinases, c-Met, claudin 7, CD166, VEGF mRNA and protein expression as well as high mitotic index, proliferative activity and aneuploidy in mononuclear stromal cells [18,19,20,21,22]. Nevertheless, these biomarkers of GCTB progression have been assessed in small case series only, and they have not yet been included in routine diagnostic practice.

## 2. Materials and Methods

### 2.1. Tumor Samples

Tumor tissue samples were obtained from patients suffering from GCTB. The Research Ethics Committee of St. Anne’s University Hospital (Brno, Czech Republic) approved the study protocol, and written informed consent was obtained from each participant prior to their enrollment in this study. The samples included in this study were taken from thirteen patients (9 males and 4 females; age range: 16–51 years old); a description of the cohort of patients is provided in Table 1. Eight patients were treated with denosumab, six of whom underwent surgery immediately after 3–6 months of treatment. In one case, sunitinib was added to denosumab treatment for one month. Two patients underwent surgery 11 or 16 months after denosumab treatment due to relapse of disease.

### 2.2. Cell Lines and Cell Culture

Nine cell lines (Table 1) derived from respective biopsy samples that were taken from patients surgically treated for GCTB were established in our laboratory according to the previously published procedure [17]. Cells were cultivated in Dulbecco’s modified Eagle’s medium (DMEM) supplemented with 20% fetal calf serum, 2 mM glutamine, 100 IU/mL penicillin and 100 µg/mL streptomycin (all purchased from GE Healthcare Europe GmbH, Freiburg, Germany). The cell lines were maintained under standard conditions at 37 °C in a humidified atmosphere containing 5% CO_2_ and were subcultured one or two times per week.

### 2.3. Evaluation of Tissue and Cell Morphology

Tumor tissue samples were fixed in 10% formalin and embedded in paraffin to obtain FFPE blocks. Tissue sections (4 µm thick) were stained with hematoxylin–eosin using an automated Medite TST 44 slide stainer (Medite Medical, Burgdorf, Germany) and mounted on glass slides. Cultured cells growing on glass coverslips were rinsed in PBS and fixed in a 1:1 methanol to PBS mixture for 2 min at room temperature (RT) and then in methanol only for 10 min at RT. The cells were subsequently dried and stained with an undiluted Giemsa stain for 2 min and with Giemsa diluted in water (1:4) for 2 min at RT. Coverslips were rinsed in water, dried and mounted onto glass slides.

### 2.4. Immunoblotting

Protein lysates were loaded onto 10% SDS-polyacrylamide gels (10 µg/well), electrophoresed and blotted onto PVDF membranes (Bio-Rad Laboratories GmbH, Munich, Germany). The membranes were then blocked with 5% solution of dry nonfat milk in PBS with 0.05% Tween 20 (PBS-Tween). Afterwards, the membranes were incubated with primary antibodies: anti-Histone H3.3 G34W (RM263, RevMAb Biosciences, South San Francisco, CA, USA), anti-total Histone H3 (1B1B2, Cell Signaling Technology, Danvers, MA, USA) and GAPDH (14C10, Cell Signaling Technology, Danvers, MA, USA). This was conducted overnight at 4 °C, followed by rinsing with PBS-Tween. The membranes were then incubated with corresponding secondary antibodies for 1 h at RT, after which the membranes were rinsed again with PBS-Tween. Chemiluminescence was induced using Amersham™ ECL™ Prime Western Blotting Detection Reagent (GE Healthcare, Little Chalfont, UK) and detected by the Azure c600 imaging system (Azure Biosystems, Dublin, CA, USA).

### 2.5. Phosphoprotein Array Analysis

The relative phosphorylation levels of tyrosine residues in 49 RTKs were analyzed using the Proteome Profiler Human Phospho-RTK Array Kit (R&D Systems, Minneapolis, MN, USA). The relative phosphorylation levels of specific phosphosites in 43 human kinases and total amounts of 2 related proteins were determined using the Proteome Profiler Human Phospho-Kinase Array kit (R&D Systems) as previously described [23]. All detected proteins are listed in the Supplementary Materials (Tables S1 and S2). The levels of phosphorylation or expression were quantified using ImageJ software (NIH, Bethesda, MD, USA) [24].

### 2.6. Principal Component Analysis

Principal component analysis (PCA) was used to classify the results of both arrays with acquired data used as variables. Data were first scaled and centered and then distributed to the first two components using the *factoextra* package [25] in R Project software [26]. Points correspond to RTKs and downstream kinases, while the contribution of tumor samples is shown as vectors.

### 2.7. MTT Assay

The proliferative activity of the GCTB-derived cell lines was determined by the MTT assay, evaluating the growth inhibitory effects of monoclonal antibody treatment or small-molecule inhibitors at three different concentrations: denosumab (30, 60 and 120 μg/mL), erlotinib (0.1, 1 and 10 μM) and sunitinib (0.1, 1 and 10 μM). For combination treatment, the cells were treated with the highest dose of denosumab (120 μg/mL) concomitantly with three different concentrations of erlotinib or sunitinib (0.1, 1 and 10 μM). A total of 2 × 10^3^ cells were seeded in 200 μL of DMEM into each well of 96-well microplates, and the cells were allowed to adhere overnight. The following day, the medium was replaced with fresh medium containing denosumab and/or small-molecule inhibitor treatment, and the microplates were incubated under standard conditions. After the intended treatment period, the medium was replaced with 200 μL of fresh DMEM containing 3-(4-dimethylthiazol-2-yl)-2,5-diphenyltetrazolium bromide (MTT, Sigma-Aldrich, St. Louis, MO, USA) at a concentration of 0.5 mg/mL. The cells were then incubated at 37 °C for 3.5 h. Following medium removal, 200 μL of DMSO was added to each well to dissolve the formazan crystals. Absorbance was then measured at 570 nm using a Sunrise Absorbance Reader (Tecan, Männedorf, Switzerland) with a reference absorbance at 620 nm.

### 2.8. Statistical Analysis

Quantitative data from the MTT assays are shown as the mean ± standard deviation (SD). Data were analyzed using one-way ANOVA, followed by the Scheffé post hoc test: * *p* < 0.001 indicates significant differences from the control. Data from phosphoprotein arrays were analyzed using the Mann–Whitney U test.

## 3. Results

### 3.1. Denosumab-Treated Tumor Samples Exhibit Specific Profiles of Phosphorylated Kinases

**Figure 1:**
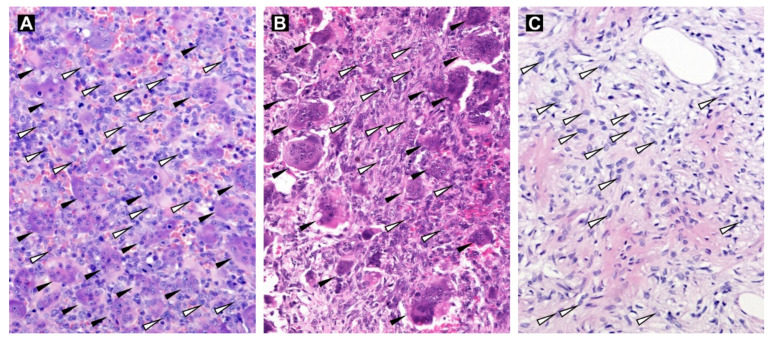
Hematoxylin-eosin-stained tissue sections of GCTB samples. Tumor tissue without denosumab treatment (Sample No. 4; **A**) and tissue of a relapsed sample (Sample No. 1; **B**) with osteoclast-like multinucleated giant cells (black arrowheads) and mononuclear stromal cells (white arrowheads; examples only). GCTB sample after denosumab treatment without the presence of osteoclast-like giant cells (Sample No. 3; **C**). Original magnification, 200×.

Five samples (Nos. 2, 3, 8, 10 and 13) from patients obtained immediately after denosumab treatment, two samples (Nos. 1 and 5) from relapsed denosumab-treated patients and five samples (Nos. 4, 6, 7, 9 and 11) from patients not treated with denosumab were used in this study. All patients were diagnosed with Campanacci grade 2 or 3 tumors, and these tumors were localized at typical sites (Table 1). At the histological level, all tumor samples without denosumab treatment (Figure 1A) and both relapsed samples obtained a relatively long time after denosumab treatment (Figure 1B) contained two typical populations of cells, i.e., osteoclast-like giant cells and mononuclear (stromal) cells. All tumor samples obtained immediately after termination of treatment with denosumab contained only neoplastic stromal cells (Figure 1C).

**Figure 2:**
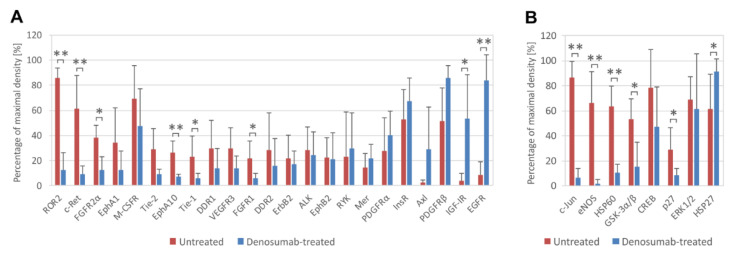
Phosphorylation analysis of RTKs and downstream signaling proteins in ten fresh-frozen GCTB samples (untreated, *n* = 5; denosumab-treated, *n* = 5). Highly phosphorylated RTKs (**A**) and downstream signaling proteins (**B**) are displayed. The cutoff level was set as 20% of maximal density. Phosphoproteins are arranged in order of differences between denosumab-treated and untreated samples. The data represent the mean ± SD. * *p* < 0.05, ** *p* < 0.01, indicating a significant difference between experimental groups.

To analyze the phosphorylation profiles of RTKs and downstream signaling proteins in denosumab-treated (Nos. 2, 3, 8, 10 and 13) and untreated (Nos. 4, 6, 7, 9 and 11) tumors, phosphoprotein arrays were employed. Our data using ten fresh-frozen tumor samples showed that in all GCTB tissues, there was similarly high relative phosphorylation of M-CSFR, InsR and PDGFRβ (Figure 2A), as well as CREB and ERK1/2 (Figure 2B), independent of the treatment.

Statistically significant increases in the phosphorylation of EGFR, IGF-IR (Figure 2A) and HSP27 (Figure 2B) were observed in samples obtained after denosumab treatment. The decrease in protein phosphorylation after treatment was more extended and affected different RTKs, e.g., ROR2, c-Ret, FGFR2α, EphA10, Tie-1 and FGFR1 (Figure 2A). Among downstream signaling proteins, the decrease in phosphorylation of c-Jun, eNOS, GSK3α/β and p27 was especially obvious. Similarly, the total level of HSP60 protein was also reduced (Figure 2B).

**Figure 3:**
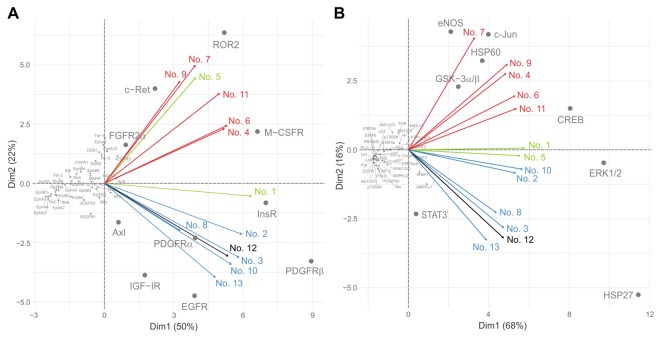
PCA of data determined for RTKs (**A**) and downstream kinases (**B**). Red vectors: samples from patients not treated with denosumab (Nos. 4, 6, 7, 9 and 11); blue vectors: samples from patients obtained immediately after denosumab treatment (Nos. 2, 3, 8, 10 and 13); green vectors: samples from relapsed denosumab-treated patients (Nos. 1 and 5); black vector: sample from patient treated with denosumab and sunitinib simultaneously (No. 12).

PCA utilizing a linear transformation of data variability was used to classify the results of both protein arrays with acquired data used as variables. A distribution of RTKs (Figure 3A) in the space of the first two principal components describes 72% of the data variability. Most kinases form a large cluster at the intersection of axes, meaning that they do not significantly contribute to the data distribution within two-dimensional space. However, there are two distinct groups of kinases separated from the main cluster that are further divided between themselves along the second principal component axis. The first group consists of FGFR2α, c-Ret, ROR2 and M-CSFR, while the second group is formed by Axl, IGF-IR, PDGFRα, EGFR, InsR and PDGFRβ receptors. These proteins were previously demonstrated to be significant in the two groups of samples. While the first group of receptors (Figure 3A, upper right quadrant) was predominantly phosphorylated in samples of untreated patients, the second group (Figure 3A, lower right quadrant) was coupled to the samples of denosumab-treated patients. This distribution is further confirmed by loading vectors that correspond to samples and describe how strongly RTKs contribute to both principal components. The closer the vectors are to each other, the more similar the tissue samples are. Figure 3B shows that the results for downstream signaling proteins described 84% of the data variability and further confirmed the patient distribution, with GSK-3α/β, HSP60, eNOS, c-Jun and CREB kinases being significant for samples from untreated patients (Figure 3B, upper right quadrant), while STAT3^I^, ERK1/2 and HSP27 kinases were dominant in the denosumab-treated group (Figure 3B, lower right quadrant). Additionally, Figure 3B shows a clear distinction of samples obtained from relapsed Patient Nos. 1 and 5.

### 3.2. GCTB-Derived Cell Lines Resemble Denosumab-Treated Cancer Tissues Regarding RTK Phosphorylation Profiles

**Figure 4:**
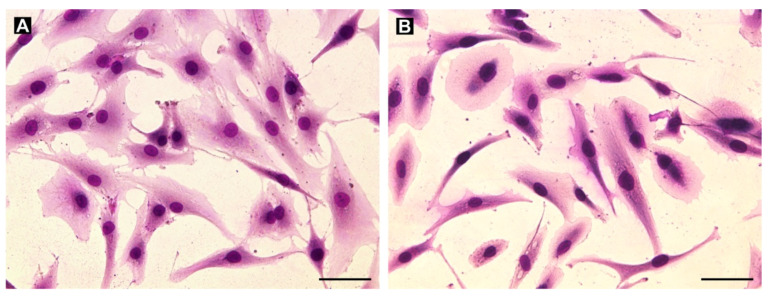
Examples of the morphology of cell lines derived from denosumab-treated (**A**, cell line GCTB8) and untreated (**B**, cell line GCTB7) samples as visualized using Giemsa staining. Mainly mononuclear (stromal) cells with fibroblast-like morphology were derived from the tumor tissues. Bar, 100 µm.

To establish an *in vitro* model of GCTB, we derived nine cell lines from the patient tumor tissues and subsequently characterized the cultured cells. Giemsa staining of all patient-derived cell lines showed the presence of mostly mononuclear (stromal) cells with a fibroblast-like morphology over a few passages (Figure 4). Moreover, regarding cell morphology, no differences between cell lines derived from samples obtained from patients with (Figure 4A) or without denosumab treatment (Figure 4B) were found.

**Figure 5:**
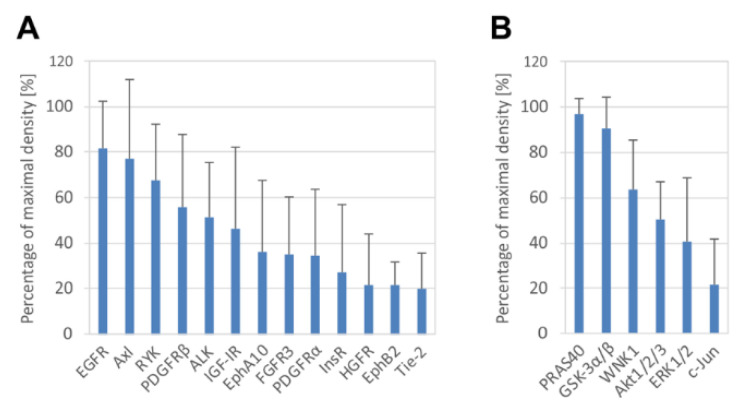
Phosphorylation analysis of RTKs and downstream signaling proteins in GCTB-derived cell lines. Highly phosphorylated RTKs (**A**) and downstream signaling proteins (**B**) are displayed. The cutoff level was set as 20% of maximal density. Phosphorylated proteins are arranged in order of percentage of maximal density. The data represent the mean of values of five (**A**) or six (**B**) analyzed cell lines ± SD.

Our phosphoprotein array experiments with GCTB-derived cell lines cultivated under standard conditions revealed high levels of several phosphorylated RTKs. Among 49 screened receptors, EGFR, Axl, RYK, PDGFRβ, ALK and IGF-IR displayed the highest levels of phosphorylation (Figure 5A). Notably, four of these proteins (i.e., EGFR, IGF-IR, PDGFRβ and Axl) also showed the highest increase in phosphorylation in cancer tissue samples after denosumab treatment compared to untreated samples (Figure 2A). This result suggests that GCTB-derived cell lines resemble denosumab-treated cancer tissues regarding RTK phosphorylation. Phosphorylation profiles of downstream signaling proteins in cell lines (Figure 5B) were completely different from those of both treated and untreated tissue samples (Figure 2B). GCTB-derived cells under *in vitro* conditions apparently utilize AKT signaling to maintain their viability in cell cultures. Apart from AKT, three AKT downstream signaling targets, PRAS40, GSK3α/β and WNK1, were also phosphorylated.

### 3.3. GCTB-Derived Cell Lines Are Sensitive to Tyrosine Kinase Inhibitors but Not to RANKL Inhibitor

Based on the results obtained using phosphoprotein arrays, we presumed that highly phosphorylated RTKs and downstream proteins were involved in the maintenance of cell viability and proliferative activity [27], and therefore, these proteins could serve as targets for treatment with low-molecular-weight inhibitors.

**Figure 6:**
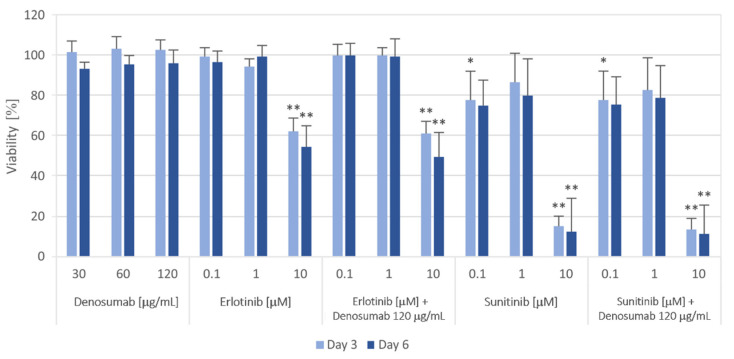
Proliferation of GCTB cell lines after 3 and 6 days of the selected treatment. Cell viability was measured using the MTT assay on Days 3 and 6 of cultivation with different concentrations of the RANKL inhibitor denosumab, EGFR inhibitor erlotinib and PDGFR inhibitor sunitinib or their combinations. The values were compared with those of untreated control cells (untreated controls were set as 100%). The data represent the mean of values of nine analyzed cell lines ± SD. Experiments with each individual cell line were performed in triplicate. * *p* < 0.05, ** *p* < 0.01, indicating a significant difference from the control.

First, we tested the homogeneity of GCTB cell lines using denosumab treatment in a range of 30 to 120 μg/mL (Figure 6), which should not affect the viability of mononuclear neoplastic cells because they do not express RANK [28]. While denosumab treatment did not change cell viability, all tumor cell lines treated with 10 µM EGFR inhibitor erlotinib showed a significant decrease in cell viability to approximately 50% after six days of incubation. Sunitinib, the second tyrosine kinase inhibitor known for its inhibitory effect on PDGFRs, VEGFRs, c-Kit and other receptors [29], suppressed cell viability to approximately 80% at 0.1 and 1 µM. The highest sensitivity of GCTB cell lines was observed at a 10 µM concentration of sunitinib, and the viability decreased to approximately 10% of the control after six days of treatment. Moreover, the effects of erlotinib or sunitinib were not modified by therapeutic concentrations of denosumab (Figure 6).

### 3.4. From Bench to Bedside: The Use of Sunitinib in GCTB Treatment

According to *in vitro* studies performed on nine GCTB-derived cell lines where the application of sunitinib resulted in a massive decrease in cell viability, we decided to use off-label treatment with sunitinib in a GCTB-diagnosed patient.

**Figure 7:**
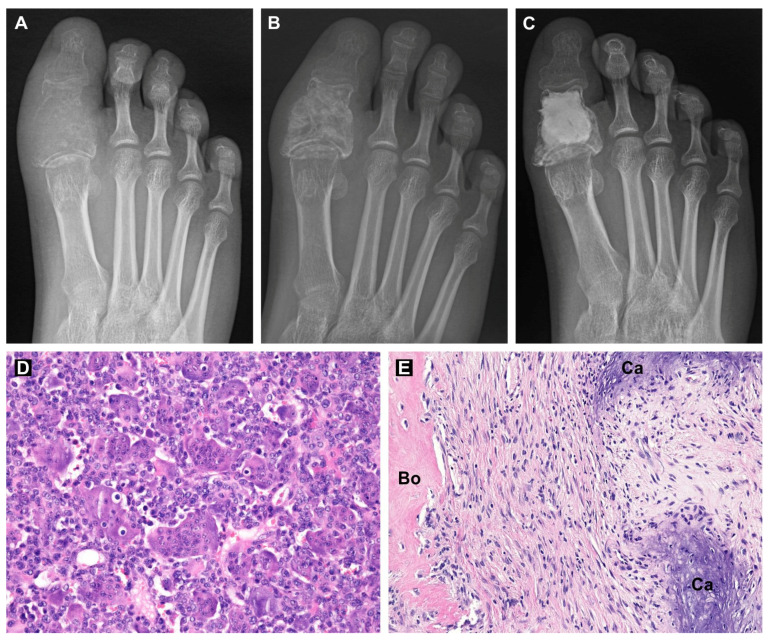
X-ray images of the patient’s right big toe during the treatment: before treatment (**A**), one month after combination treatment with denosumab and sunitinib (**B**) and after curettage and bone cavity filling with bone cement (**C**). Hematoxylin-eosin-stained sections of GCTB tissue before treatment (**D**) and after combination treatment with denosumab and sunitinib (**E**). Regions of bone tissue (Bo) and calcifications (Ca) are labeled. Original magnification, 200× (**D**,**E**).

This patient (male, 17 years old) reported right toe pain since June 2019. He observed worsening pain when at rest and increasing swelling of the toe. Three months later, the patient visited a physician, where he underwent an X-ray with a suspected bone tumor of the proximal phalanx of the big toe (Figure 7A). Then, computed tomography was performed, which showed destruction of the cortex of the proximal phalanx and the extraosseous component of the tumor. The patient was then sent to the First Department of Orthopedic Surgery at St. Anne’s University Hospital Brno, which is the center for the treatment of musculoskeletal tumors in the Czech Republic. We took a tissue sample from the tumor. Histologic examination revealed numerous multinucleated giant cells that were uniformly scattered in a background of mononuclear oval or plump stromal cells, confirming the diagnosis of giant-cell tumor of bone (Figure 7D).

Denosumab treatment was indicated due to the local progression of the tumor (Campanacci grade 3). Due to the risk of toe mutilation or toe loss during radical surgery, we decided to add sunitinib to the standard of care, e.g., neoadjuvant denosumab scheduled for three cycles, each lasting 28 days. After two cycles of denosumab treatment (given at Days 1, 8 and 15 of Cycle 1 and Day 1 of additional two cycles at a dose of 120 mg/dose s.c. injection with daily supplemented calcium and cholecalciferol), sunitinib (25 mg p.o. daily) was added to the combination for one month (Cycle 3 of denosumab). This combination treatment was discussed with the patient and his parents and was administered upon signed informed consent. The treatment was administered in an outpatient setting under supervision of a pediatric oncologist experienced in sunitinib treatment in the Department of Pediatric Oncology, University Hospital Brno. No severe adverse events were observed. The patient experienced only a mild dysesthesia as a symptom of hypocalcemia after the first dose of denosumab and temporary hand-foot erythema during the fifth week of denosumab administration. Control X-ray and MR were then performed, showing tumor regression and disappearance of the soft tissue component (Figure 7B).

After the termination of the neoadjuvant treatment, we performed curettage using a helium beam and filled the bone cavity with bone cement (Figure 7C). The surgical specimen showed variable cellular tissue resembling scar in various stages of maturation. Irregular trabeculae of woven bone and areas of coarse calcifications were haphazardly distributed throughout the tumor. There was a complete depletion of multinucleated giant cells and mononuclear stromal cells (Figure 7E). The absence of stromal cells was also confirmed by immunohistochemistry. The tissue sample was p63 and histone H3.3 G34W negative. Three adjuvant cycles of denosumab and sunitinib were administered following limb salvage surgery. To date (6/2021), the patient is 16 months postoperation, with full mobility of the toe, no problems and no signs of local recurrence of the tumor.

## 4. Discussion

The preferred treatment option for GCTB is curettage with the use of local adjuvant therapy (phenol, argon beam and bone cement). In the case of tumors with soft tissue extension, *en bloc* resection should be performed [6,13,30]. The use of denosumab is indicated in cases when radical resection cannot be performed and for the control of metastatic lung disease [4,12]. A reduction in tumor size, a calcified rim around the tumor and tumor soft tissue components were observed after three months of therapy. Neoadjuvant denosumab may cause downstaging of the tumor and facilitate *en bloc* resection [11,12,30]. However, the risk of GCTB recurrence is still relatively high and dependent on the age at diagnosis. Patients under 25 years of age have the greatest risk of local recurrence [31]. The mechanism of recurrence is based on persistent neoplastic stromal cells, which do not respond to denosumab treatment [14,15] and likely maintain their proliferative ability. The termination of treatment could lead to overexpression of RANKL and RANK by neoplastic stromal cells and osteoclast-like giant cells, respectively [32]. For this reason, we focused on cell signaling in tumor tissue and in tumor-derived cell lines to identify druggable targets in the neoplastic component of GCTB tissue.

Although previous studies have reported that denosumab does not affect mononuclear tumor cells [14,15,33], our results demonstrated the effects of denosumab treatment on phosphorylation profiles in GCTB tissue. The tumor tissue samples before denosumab treatment showed a specific pattern of RTK and downstream protein phosphorylation. Among RTKs, there was increased phosphorylation of M-CSFR, InsR, ROR2, c-Ret and PDGFRβ, whose expression is directly associated with GCTB promotion [34,35] and osteoblast or osteoclast differentiation [36,37,38]. Similar processes related to bone remodeling are also associated with the highly phosphorylated downstream signaling proteins c-Jun, eNOS, HSP60, GSK3α/β, CREB, ERK1/2 and HSP27 [39,40,41,42,43,44]. This specific signaling profile typical of untreated GCTB tissue is subject to change after denosumab treatment. Moreover, the principal component analysis of protein array data confirms the separation of two groups of patients and their respective samples that were and were not subjected to denosumab treatment.

On the histological level, denosumab causes a significant reduction in the number of osteoclast-like giant cells [45]; therefore, the signaling profiles of denosumab-treated GCTB samples characterize the remaining populations of mononuclear neoplastic stromal cells. It is possible to assume that downregulated proteins after denosumab treatment play an important role in GCTB tissue with both giant and stromal interacting cell types, while stable or upregulated proteins are predominantly expressed in stromal cells. Two proteins, EGFR and PDGFRβ, also showed high phosphorylation in GCTB tissue-derived cell lines. EGFR expression was found in neoplastic stromal cells, mainly in recurrent GCTB tissues, where EGFR signaling may contribute to disease progression by promoting stromal cell proliferation and osteoclastogenesis [18]. Similarly, high expression of PDGFRβ mRNA was detected in GCTB tissue [46], and the low-molecular-weight inhibitor imatinib was proven to inhibit proliferation of osteoblastic cells via the PDGFR signaling [47]. Moreover, the inhibition of PDGFR signaling led to increased production of osteoprotegerin by osteoblastic/stromal cells, which reduced RANKL/RANK signaling [48].

Based on the data obtained, we selected the EGFR inhibitor erlotinib and the PDGFR inhibitor sunitinib as suitable tyrosine kinase inhibitors for the *in vitro* experiments in this study. Erlotinib is indicated for the treatment of patients with metastatic non-small-cell lung cancer, locally advanced, unresectable or metastatic pancreatic cancer and malignant gliomas [49,50]. Sunitinib is a multitargeted receptor tyrosine kinase inhibitor that is representative of antiangiogenic drugs and has been approved for the treatment of GIST and renal cell carcinoma [51,52].

All cell lines treated with tyrosine kinase inhibitors (sunitinib or erlotinib) showed a significant reduction in cell viability that was not modified by therapeutic concentrations of denosumab. A higher sensitivity of GCTB cell lines was observed using sunitinib, and its effectivity was approximately five times higher than erlotinib at a concentration of 10 µM. This finding is consistent with the results of wide anticancer drug screening on an established GCTB cell line, where PDGFR inhibitors reduced cell viability more effectively than EGFR inhibitors [53]. To date, there is only one multicenter phase II trial with sunitinib in the treatment of non-GIST sarcoma. Although only one patient with advanced GCTB was present in the experiment, it was a stable disease for at least 68 weeks [52]. One case of an adolescent patient with metastases of a giant-cell tumor of bone to the lungs who was treated with denosumab and sunitinib has also been reported in the literature. According to the results of tumor gene testing (high expression of PDGFR and VEGFR), sunitinib was added to the treatment strategy, and lung metastases were reduced within 5 months of treatment, but there were no post-treatment evaluations of the tumor sample in this case, and complete remission was not achieved [44].

In our case, a 17-year-old patient with Campanacci grade 3 GCTB of the big toe was at risk of whole toe amputation. Denosumab treatment was indicated due to the local progression of the tumor [12]. Based on our *in vitro* results, which were pending during the initial two cycles of denosumab monotherapy, sunitinib (25 mg p.o. daily) was added to the combination for the last month of neoadjuvant treatment before definitive surgery. Control imaging methods showed regression and bordering of the tumor. Then, we performed curettage using a helium beam and filled the bone with cement. Histologic examination revealed scar-like tissue with the proliferation of cytologically uniform spindle cells in a background of collagenous matrix. There was a complete depletion of multinucleated giant cells and mononuclear stromal cells. Treatment with denosumab led to the disappearance of all giant osteoclast-like cells in the tumor tissue [14,17,54], implying that sunitinib contributed to the suppression of stromal tumor cells. Interestingly, such a complete response has been achieved with sunitinib at the recommended dose for the treatment of malignancies according to the approved product label.

Close monitoring of adverse events was performed during combination treatment, including regular screening for echocardiogram and electrocardiogram abnormalities, as known adverse events of sunitinib are heart failure and QTc interval prolongation. The latter is of special interest because possible hypocalcemia after denosumab can be an additional risk factor for malignant arrhythmia. Thus, normocalcemia should be maintained during combination treatment, and the patient should be instructed to report and immediately manage all minimal signs of hypocalcemia. At present, the patient is 15 months postsurgery, has no problems and there are no signs of local recurrence of the tumor or any toxicity of combination therapy. There is no consensus on the length of treatment with denosumab in the neoadjuvant or adjuvant setting [55].

Taken together, two main effects of PDGFR signaling and its inhibition concerning bone remodeling and GCTB progression could be considered. First, PDGF-BB/PDGFRβ signaling stimulates osteoprogenitors and osteoblast proliferation and migration and inhibits their maturation *in vitro* [38]. Second, the blocking of PDGF/PDGFR signaling increased gene expression and protein secretion of OPG in stromal and osteoblastic cells [48]. Therefore, the possible outcomes of the treatment with TKI sunitinib are: (i) direct inhibition of the proliferation of stromal cells as we detected in cell lines under *in vitro* conditions; (ii) differentiation of stromal cells to fibroblast-like cells as we detected in tumor tissue samples; (iii) indirect inhibition of RANKL/RANK signaling in giant cells as described by O’Sullivan and colleagues [48].

## 5. Conclusions

In summary, denosumab inhibited osteoclast-like giant cells and altered the phosphorylation profiles of receptor tyrosine kinases and downstream signaling proteins in tumor tissue. Analysis of GCTB tissue samples and GCTB-derived cell lines revealed PDGFRβ as a suitable target for treatment with FDA-approved small-molecule inhibitors. Sunitinib, a PDGFR inhibitor, was responsible for a significant reduction in the viability of tumor stromal cells under *in vitro* conditions, similar to tumor tissue. Treatment of GCTB with denosumab in combination with sunitinib could become an effective precision treatment in locally advanced tumors and metastatic disease due to its role in targeting both main cell populations composing the tumor tissue. Taken together, our *in vitro* and in vivo data show that a specific RTK signaling pattern is activated in GCTB cells and plays an important role in the regulation of cell proliferation. Thus, some RTKs and their downstream signaling pathways represent useful targets for precision treatment with low-molecular-weight inhibitors or with other types of modern biological therapy. Nevertheless, these promising results should be verified using a large number of GCTB samples, and confirmation of successful combination treatment should be explored in similar patients at risk of limb mutilation or limb loss due to recurrent GCTB.

## Figures and Tables

**Table 1:** Overview of the patients and samples included in this study.

Sample Number	Sex	Age at Diagnosis	Localization	Campanacci Grade	Denosumab Treatment	Cell Line
1	M	33	Proximal tibia	3	Yes ^†^	GCTB1
2	M	46	Proximal ulna	3	Yes	Not derived
3	M	27	Distal radius	3	Yes	GCTB3
4	M	37	Distal radius	3	No	GCTB4
5	F	46	Distal femur	2	Yes *	GCTB5
6	M	25	Proximal fibula	3	No	GCTB6
7	M	41	Distal femur	2	No	GCTB7
8	M	28	Cuboid bone	3	Yes	GCTB8
9	F	45	Proximal tibia	2	No	GCTB9
10	F	16	Proximal fibula	3	Yes	GCTB10
11	F	51	Proximal tibia	2	No	Derived but not used
12	M	17	Proximal phalanx of big toe	3	Yes ^#^	Not derived
13	M	40	Distal radius	2	Yes	Derived but not used

^†^ patient was treated with denosumab 11 months before surgery, * patient was treated with denosumab 16 months before surgery, ^#^ patient was treated with denosumab and sunitinib simultaneously for 1 month. Campanacci grades: Grade 2 lesions have a relatively well-defined margin but no radiopaque rim, and the cortical bone is thinned; Grade 3 lesions have indistinct borders with cortical destruction and soft tissue extension.

## Data Availability

The data presented in this study are available in the article and the Supplementary Materials.

## Supplementary Materials

The following are available online at https://www.mdpi.com/article/10.3390/cancers13143543/s1, Figure S1: Analysis of mutated 466 histone H3.3G34W and total histone H3 expression in cell lines by immunoblotting, Figure S2: Hematoxylin-eosin-stained tissue sections of GCTB samples, Table S1: Overview of phosphorylated proteins detected using Proteome Profiler™ Human Phospho-RTK Array Kit, Table S2: Overview of phosphorylated proteins including phosphorylation sites detected using the Proteome Profiler™ Human Phospho-Kinase Array Kit.

## References

[1] Campanacci M., Baldini N., Boriani S., Sudanese A. (1987). Giant-Cell Tumor of Bone. J. Bone Joint Surg. Am..

[2] Athanasou N., Bansal M., Forsyth R., Reid R., Sapi Z., Fletcher C.D.M., Bridge J., Hogendoor P., Mertens F. (2013). Giant cell tumor of bone. WHO Classification of Tumours of Soft Tissue and Bone.

[3] Turcotte R.E. (2006). Giant Cell Tumor of Bone. Orthop. Clin. North Am..

[4] Unni K.K., Inwards C.Y. (2009). Dahlin’s Bone Tumors: General Aspects and Data on 10,165 Cases.

[5] Ruggieri P., Mavrogenis A.F., Ussia G., Angelini A., Papagelopoulos P.J., Mercuri M. (2010). Recurrence After and Complications Associated With Adjuvant Treatments for Sacral Giant Cell Tumor. Clin. Orthop. Relat. Res..

[6] Balke M., Schremper L., Gebert C., Ahrens H., Streitbuerger A., Koehler G., Hardes J., Gosheger G. (2008). Giant cell tumor of bone: Treatment and outcome of 214 cases. J. Cancer Res. Clin. Oncol..

[7] Malek F.A.A., Krueger P.M., Hatmi Z.N., Malayeri A., Faezipour H., Donnell R.J.O. (2006). Local control of long bone giant cell tumour using curettage, burring and bone grafting without adjuvant therapy. Int. Orthop..

[8] Saiz P., Virkus W., Piasecki P., Templeton A., Shott S., Gitelis S. (2004). Results of Giant Cell Tumor of Bone Treated With Intralesional Excision. Clin. Orthop. Relat. Res..

[9] Jeys L.M., Suneja R., Chami G., Grimer R.J., Carter S.R., Tillman R.M. (2006). Impending fractures in giant cell tumours of the distal femur: Incidence and outcome. Int. Orthop..

[10] Wu P.-F., Tang J.-Y., Li K.-H. (2015). RANK pathway in giant cell tumor of bone: Pathogenesis and therapeutic aspects. Tumor Biol..

[11] Thomas D.M. (2012). RANKL, denosumab, and giant cell tumor of bone. Curr. Opin. Oncol..

[12] Chawla S., Henshaw R., Seeger L., Choy E., Blay J.-Y., Ferrari S., Kroep J., Grimer R., Reichardt P., Rutkowski P. (2013). Safety and efficacy of denosumab for adults and skeletally mature adolescents with giant cell tumour of bone: Interim analysis of an open-label, parallel-group, phase 2 study. Lancet Oncol..

[13] Becker W.T., Dohle J., Bernd L., Braun A., Cserhati M., Enderle A., Hovy L., Matejovsky Z., Szendroi M., Arbeitsgemeinschaft Knochentumoren (2008). Local Recurrence of Giant Cell Tumor of Bone after Intralesional Treatment with and without Adjuvant Therapy. J. Bone Jt. Surg. Am. Vol..

[14] Rutkowski P., Gaston L., Borkowska A., Stacchiotti S., Gelderblom H., Baldi G.G., Palmerini E., Casali P., Gronchi A., Parry M. (2018). Denosumab treatment of inoperable or locally advanced giant cell tumor of bone—Multicenter analysis outside clinical trial. Eur. J. Surg. Oncol. EJSO.

[15] Palmerini E., Chawla N., Ferrari S., Sudan M., Picci P., Marchesi E., Leopardi M.P., Syed I., Sankhala K., Parthasarathy P. (2017). Denosumab in advanced/unresectable giant-cell tumour of bone (GCTB): For how long?. Eur. J. Cancer.

[16] Ulas A., Akinci M.B., Silay K., Sendur M.A.N., DeDe D.S., Yalcin B. (2015). Denosumab: Excellent response of metastatic giant cell tumor of the bone. J. BUON Off. J. Balk. Union Oncol..

[17] Mak I.W., Evaniew N., Popovic S., Tozer R., Ghert M. (2014). A Translational Study of the Neoplastic Cells of Giant Cell Tumor of Bone Following Neoadjuvant Denosumab. J. Bone Jt. Surg. Am. Vol..

[18] Balla P., Moskovszky L., Sapi Z., Forsyth R., Knowles H., Athanasou N.A., Szendroi M., Kopper L., Rajnai H., Pinter F. (2011). Epidermal growth factor receptor signalling contributes to osteoblastic stromal cell proliferation, osteoclastogenesis and disease progression in giant cell tumour of bone. Histopathology.

[19] Guenther R., Krenn V., Morawietz L., Dankof A., Melcher I., Schaser K.-D., Kasper H.-U., Kuban R.-J., Ungethüm U., Sers C. (2005). Giant cell tumors of the bone: Molecular profiling and expression analysis of Ephrin A1 receptor, Claudin 7, CD52, FGFR3 and AMFR. Pathol. Res. Pr..

[20] Liu L., Aleksandrowicz E., Fan P., Schonsiegel F., Zhang Y., Sahr H., Gladkich J., Mattern J., Depeweg D., Lehner B. (2014). Enrichment of c-Met+ tumorigenic stromal cells of giant cell tumor of bone and targeting by cabozantinib. Cell Death Dis..

[21] Zhou Z., Li Y., Wang X., Hu J., Kuang M., Wang Z., Li S., Xu W., Xiao J. (2018). ALCAM+ stromal cells: Role in giant cell tumor of bone progression. Cell Death Dis..

[22] Maros M.E., Schnaidt S., Balla P., Kelemen Z., Sapi Z., Szendroi M., Laszlo T., Forsyth R., Picci P., Krenacs T. (2019). In situ cell cycle analysis in giant cell tumor of bone reveals patients with elevated risk of reduced progression-free survival. Bone.

[23] Neradil J., Kyr M., Polaskova K., Kren L., Macigova P., Skoda J., Sterba J., Veselska R. (2019). Phospho-Protein Arrays as Effective Tools for Screening Possible Targets for Kinase Inhibitors and Their Use in Precision Pediatric Oncology. Front. Oncol..

[24] Schneider C.A., Rasband W.S., Eliceiri K.W. (2012). NIH Image to ImageJ: 25 years of image analysis. Nat. Meth..

[25] Kassambara A., Mundt F. (2020). Factoextra: Extract and Visualize the Results of Multivariate Data Analyses. R package Version 1.0.7. https://CRAN.R-project.org/package=factoextra.

[26] R Core Team (2018). R: A Language and Environment for Statistical Computing. https://www.R-project.org.

[27] Sramek M., Neradil J., Macigova P., Mudry P., Polaskova K., Slaby O., Noskova H., Sterba J., Veselska R. (2018). Effects of Sunitinib and Other Kinase Inhibitors on Cells Harboring a PDGFRB Mutation Associated with Infantile Myofibromatosis. Int. J. Mol. Sci..

[28] Mukaihara K., Suehara Y., Kohsaka S., Akaike K., Tanabe Y., Kubota D., Ishii M., Fujimura T., Kazuno S., Okubo T. (2016). Protein Expression Profiling of Giant Cell Tumors of Bone Treated with Denosumab. PLoS ONE.

[29] Roskoski R. (2018). The role of small molecule platelet-derived growth factor receptor (PDGFR) inhibitors in the treatment of neoplastic disorders. Pharmacol. Res..

[30] Van der Heijden L., Dijkstra P.S., Blay J.-Y., Gelderblom H. (2017). Giant cell tumour of bone in the denosumab era. Eur. J. Cancer.

[31] Klenke F.M., Wenger D.E., Inwards C.Y., Rose P.S., Sim F.H. (2011). Giant Cell Tumor of Bone: Risk Factors for Recurrence. Clin. Orthop. Relat. Res..

[32] Van Langevelde K., McCarthy C.L. (2020). Radiological findings of denosumab treatment for giant cell tumours of bone. Skelet. Radiol..

[33] Thomas D.M., Skubitz K.M. (2009). Giant cell tumour of bone. Curr. Opin. Oncol..

[34] Baud’Huin M., Renault R., Charrier C., Riet A., Moreau A., Brion R., Gouin F., Duplomb L., Heymann D. (2010). Interleukin-34 is expressed by giant cell tumours of bone and plays a key role in RANKL-induced osteoclastogenesis. J. Pathol..

[35] Oreffo R.O., Marshall G.J., Kirchen M., Garcia C., Gallwitz W.E., Chavez J., Mundy G.R., Bonewald L.F. (1993). Characterization of a Cell Line Derived from a Human Giant Cell Tumor That Stimulates Osteoclastic Bone Resorption. Clin. Orthop. Relat. Res..

[36] Hadjidakis D.J., Androulakis I.I. (2006). Bone Remodeling. Ann. N. Y. Acad. Sci..

[37] Uehara S., Udagawa N., Kobayashi Y. (2019). Regulation of osteoclast function via Rho-Pkn3-c-Src pathways. J. Oral Biosci..

[38] Brun J., Andreasen C.M., Ejersted C., Andersen T.L., Caverzasio J., Thouverey C. (2020). PDGF Receptor Signaling in Osteoblast Lineage Cells Controls Bone Resorption Through Upregulation of Csf1 Expression. J. Bone Miner. Res..

[39] David J.-P., Sabapathy K., Hoffmann O., Idarraga M.H., Wagner E.F. (2002). JNK1 modulates osteoclastogenesis through both c-Jun phosphorylation-dependent and -independent mechanisms. J. Cell Sci..

[40] Boyle W.J., Simonet W.S., Lacey D.L. (2003). Osteoclast differentiation and activation. Nat. Cell Biol..

[41] Baloul S.S. (2016). Osteoclastogenesis and Osteogenesis during Tooth Movement. Front Oral Biol.

[42] Wang L., Han L., Xue P., Hu X., Wong S.-W., Deng M., Tseng H.C., Huang B.-W., Ko C.-C. (2021). Dopamine suppresses osteoclast differentiation via cAMP/PKA/CREB pathway. Cell. Signal..

[43] Roccisana J., Kawanabe N., Kajiya H., Koide M., Roodman G.D., Reddy S.V. (2004). Functional Role for Heat Shock Factors in the Transcriptional Regulation of Human RANK Ligand Gene Expression in Stromal/Osteoblast Cells. J. Biol. Chem..

[44] Koh J.-M., Lee Y.-S., Kim Y.S., Park S.-H., Lee S.H., Kim H.-H., Lee M.-S., Lee K.-U., Kim G.S. (2009). Heat shock protein 60 causes osteoclastic bone resorption via toll-like receptor-2 in estrogen deficiency. Bone.

[45] Tariq M.U., Umer M., Khan Z., Saeed J., Siddiqui M.A., Din N.U. (2020). Spectrum of histological features of Denosumab treated Giant Cell Tumor of Bone; potential pitfalls and diagnostic challenges for pathologists. Ann. Diagn. Pathol..

[46] Wang G., Jiang S., Li Z., Dong Y. (2019). Denosumab and Sunitinib in the treatment of giant-cell tumor of bone with pulmonary and bone metastases in an adolescent. Medicine.

[47] O’Sullivan S., Naot D., Callon K., Porteous F., Horne A., Wattie D., Watson M., Cornish J., Browett P., Grey A. (2007). Imatinib Promotes Osteoblast Differentiation by Inhibiting PDGFR Signaling and Inhibits Osteoclastogenesis by Both Direct and Stromal Cell-Dependent Mechanisms. J. Bone Miner. Res..

[48] O’Sullivan S., Tay M.L., Lin J., Bava U., Callon K., Cornish J., Naot D., Grey A. (2016). Tyrosine Kinase Inhibitors Regulate OPG through Inhibition of PDGFRβ. PLoS ONE.

[49] Greenhalgh J., Dwan K., Boland A., Bates V., Vecchio F., Dundar Y., Jain P., Green J.A. (2016). First-line treatment of advanced epidermal growth factor receptor (EGFR) mutation positive non-squamous non-small cell lung cancer. Cochrane Database Syst. Rev..

[50] Kelley R.K., Ko A. (2008). Erlotinib in the treatment of advanced pancreatic cancer. Biol. Targets Ther..

[51] Motzer R.J., Ravaud A., Patard J.-J., Pandha H.S., George D.J., Patel A., Chang Y.-H., Escudier B., Donskov F., Magheli A. (2018). Adjuvant Sunitinib for High-risk Renal Cell Carcinoma After Nephrectomy: Subgroup Analyses and Updated Overall Survival Results. Eur. Urol..

[52] George S., Merriam P., Maki R.G., Abbeele A.D.V.D., Yap J., Akhurst T., Harmon D.C., Bhuchar G., O’Mara M.M., D’Adamo D.R. (2009). Multicenter Phase II Trial of Sunitinib in the Treatment of Nongastrointestinal Stromal Tumor Sarcomas. J. Clin. Oncol..

[53] Noguchi R., Yoshimatsu Y., Ono T., Sei A., Hirabayashi K., Ozawa I., Kikuta K., Kondo T. (2021). Establishment and characterization of NCC-PLPS1-C1, a novel patient-derived cell line of pleomorphic liposarcoma. Hum. Cell.

[54] Basu Mallick A., Chawla S.P. (2021). Giant Cell Tumor of Bone: An Update. Curr. Oncol. Rep..

[55] Palmerini E., Staals E.L., Jones L.B., Donati D.M., Longhi A., Randall R.L. (2020). Role of (Neo)adjuvant Denosumab for Giant Cell Tumor of Bone. Curr. Treat. Options Oncol..

