# Supplementary material for: New Target for Precision Medicine Treatment of Giant-Cell Tumor of Bone: Sunitinib Is Effective in the Treatment of Neoplastic Stromal Cells with Activated PDGFRβ Signaling
Source: Cancers (Basel). 2021 Jul 15;13(14):3543. doi: 10.3390/cancers13143543 (PMC8305892; doi:10.3390/cancers13143543)
Supplement: Supplementary file 1 [file cancers-13-03543-s001.zip › cancers-1234944-supplementary.pdf]

cancers

MDPI

Supplementary Materials: New Target for Precision Medicine Treatment of Giant-Cell Tumor of Bone: Sunitinib Is Effective in the Treatment of Neoplastic Stromal Cells with Activated PDGFRβ Signaling

Michal Mahdal, Jakub Neradil, Peter Mudry, Silvia Paukovcekova, Iva Staniczkova Zambo, Jiri Urban, Peter Macsek, Lukas Pazourek, Tomas Tomas and Renata Veselska

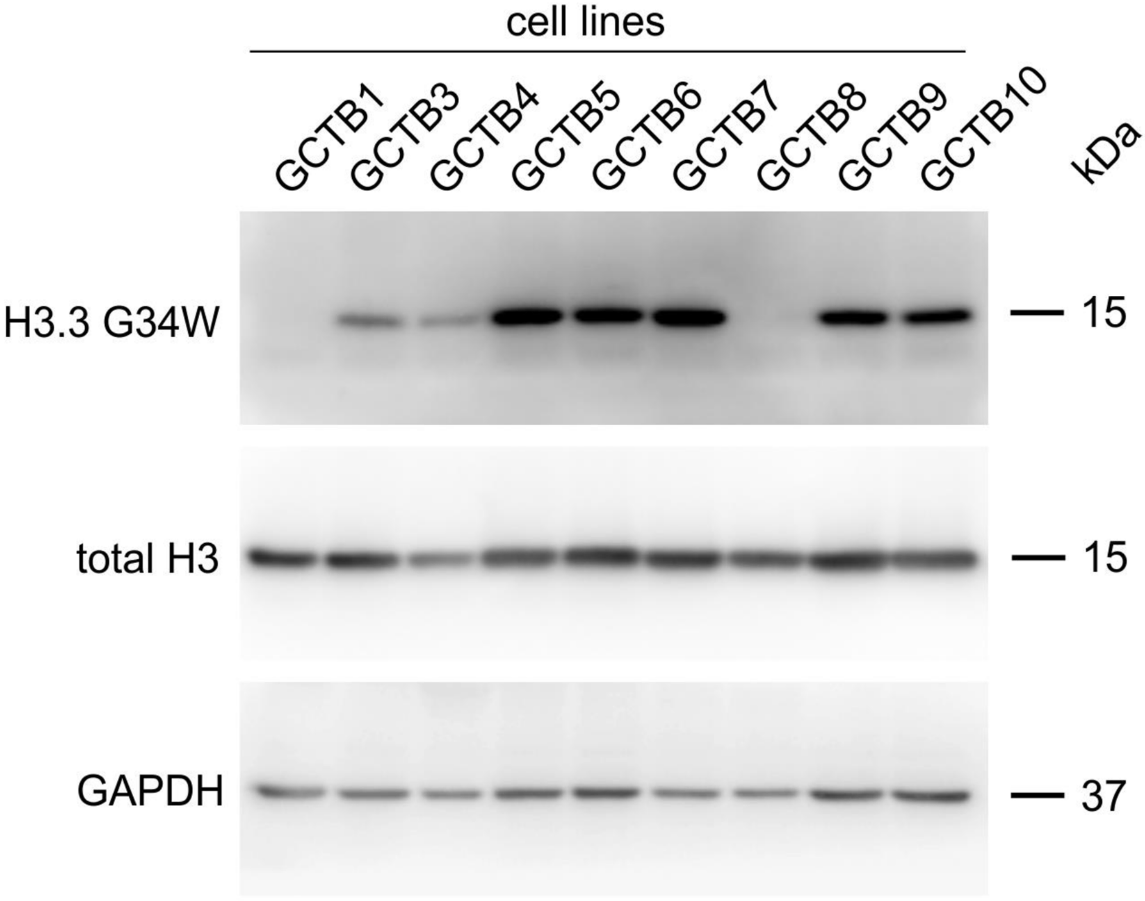

Figure S1. Analysis of mutated 466 histone H3.3G34W and total histone H3 expression in cell lines by immunoblotting.

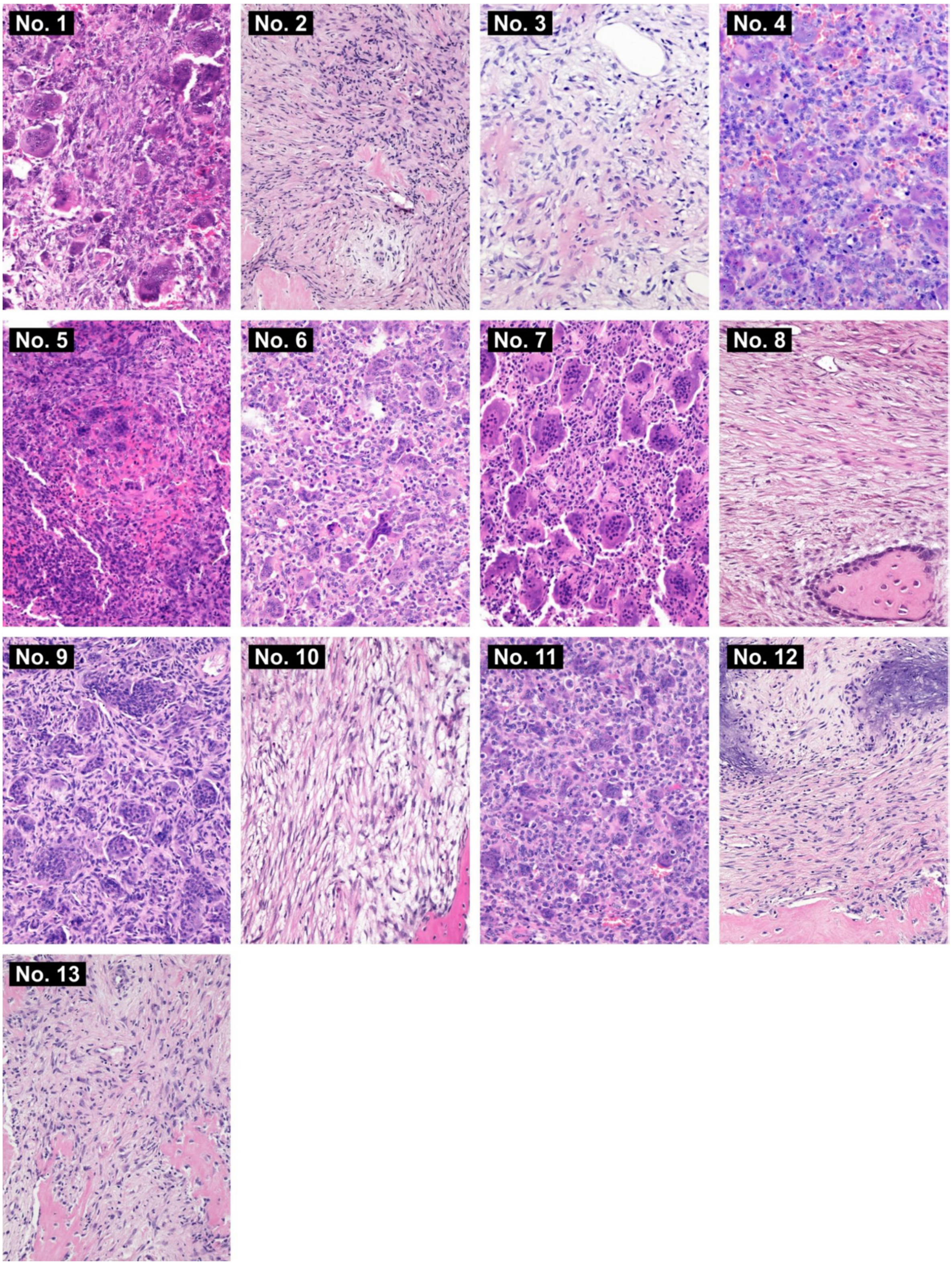

Figure S2. Hematoxylin-eosin-stained tissue sections of GCTB samples.

Table S1. Overview of phosphorylated proteins detected using the Proteome Profiler™ Human Phospho-RTK Array Kit.

EGFR	FGFR4	MSPR	ROR1	VEGFR1	EphA4	ALK
ErbB2	InsR	PDGFRα	ROR2	VEGFR2	EphA6	DDR1
ErbB3	IGF-IR	PDGFRβ	Tie-1	VEGFR3	EphA7	DDR2
ErbB4	Axl	SCFR	Tie-2	MuSK	EphB1	EphA5
FGFR1	Dtk	Flt-3	TrkA	EphA1	EphB2	EphA10
FGFR2α	Mer	M-CSFR	TrkB	EphA2	EphB4	EphB3
FGFR3	HGFR	c-Ret	TrkC	EphA3	EphB6	RYK

Table S2. Overview of phosphorylated proteins, including phosphorylation sites, detected using the Proteome Profiler™ Human Phospho-Kinase Array Kit. Upper indices are used to mark different phosphorylation sites in the same protein.

Protein	Phosphorylation site(s)	Protein	Phosphorylation site(s)
AKT1/2/3I	S473	p53I	S392
AKT1/2/3II	T308	p53II	S46
AMPKα1	T183	p53III	S15
AMPKα2	T172	p70S6KI	T389
Chk-2	T68	p70S6KII	T421/S424
c-Jun	S63	PDGFRβ	Y751
CREB	S133	PLC-γ1	Y783
EGFR	Y1086	PRAS40	T246
eNOS	S1177	PYK2	Y402
ERK 1/2	T202/Y204, T185/Y187	RSK1/2/3	S380/S386/S377
FAK	Y397	Src	Y419
Fgr	Y412	STAT2	Y689
Fyn	Y420	STAT3I	Y705
GSK3-α/β	S21/S9	STAT3II	S727
Hck	Y411	STAT5a	Y689
HSP27	S78/S82	STAT5a/b	Y694/Y699
HSP60	-	STAT5b	Y699
JNK 1/2/3	T183/Y185, T221/Y223	STAT6	Y641
Lck	Y394	TOR	S2448
Lyn	Y397	WNK1	T60
MSK 1/2	S376/S360	Yes	Y426
p27	T198	β-catenin	-
p38α	T180/Y182		
